# Molecular Imaging in Traditional Chinese Medicine Therapy for Neurological Diseases

**DOI:** 10.1155/2013/608430

**Published:** 2013-10-07

**Authors:** Zefeng Wang, Haitong Wan, Jinhui Li, Hong Zhang, Mei Tian

**Affiliations:** ^1^Department of Nuclear Medicine, The Second Affiliated Hospital of Zhejiang University School of Medicine, 88 Jiefang Road, Hangzhou, Zhejiang 310009, China; ^2^Zhejiang University Medical PET Center, Zhejiang University, Hangzhou 310009, China; ^3^Institute of Nuclear Medicine and Molecular Imaging, Zhejiang University, Hangzhou 310009, China; ^4^Key Laboratory of Medical Molecular Imaging of Zhejiang Province, Hangzhou 310009, China; ^5^Institute of Cardiocerbrovascular Diseases, Zhejiang Chinese Medical University, Hangzhou 310053, China

## Abstract

With the speeding tendency of aging society, human neurological disorders have posed an ever increasing threat to public health care. Human neurological diseases include ischemic brain injury, Alzheimer's disease, Parkinson's disease, and spinal cord injury, which are induced by impairment or specific degeneration of different types of neurons in central nervous system. Currently, there are no more effective treatments against these diseases. Traditional Chinese medicine (TCM) is focused on, which can provide new strategies for the therapy in neurological disorders. TCM, including Chinese herb medicine, acupuncture, and other nonmedication therapies, has its unique therapies in treating neurological diseases. In order to improve the treatment of these disorders by optimizing strategies using TCM and evaluate the therapeutic effects, we have summarized molecular imaging, a new promising technology, to assess noninvasively disease specific in cellular and molecular levels of living models in vivo, that was applied in TCM therapy for neurological diseases. In this review, we mainly focus on applying diverse molecular imaging methodologies in different TCM therapies and monitoring neurological disease, and unveiling the mysteries of TCM.

## 1. Introduction

With the dramatic improvement of average life expectancy and increasing trend of the aged population in recent years, neurological diseases have become a major problem of public health. Human neurological diseases such as ischemic brain injury, Alzheimer's disease (AD), Parkinson's disease (PD), depression, and spinal cord injury are caused by loss and impairment of different types of neurons in central nervous system. Current therapies, mainly focusing on western therapy such as interventional procedures, surgery, and synthetic drug, are limited in their ability to improve neural function because they fail to repair damaged neurons or improve neural regeneration. With intense exchanges between the eastern and western of the world, traditional Chinese medicine (TCM) becomes more popular and is progressively recognized all over the word. Here we mainly focus on using TCM treatment against neurological disorders.

TCM is a unique system to diagnose and cure illness, and TCM has been widely investigated, especially in China [1]. TCM is practiced in the Chinese health care system for more than 2,000 years. The Chinese have accumulated significant experience in disease prevention, diagnosis, and treatment and formed a holistic system of medicine and therapy through continuous attempts and practice for generations. TCM theories study the physiologic activities and pathologic changes of the human body and their inner interrelationships according to the phenomena and laws of nature. The clinical diagnosis and treatment begins with the analysis of the whole body system, focusing on correcting pathologic changes through adjusting the functions of the five organs (heart, liver, spleen, lung, and kidney). However, the mysteries of TCM have not been totally understood in modern society.

With developing of molecular imaging technology in the early 21st century, the unique theoretical system of medicine and therapy gradually was elaborated. Molecular imaging generally was defined as the visual representation, characterization, and quantification of biological processes at the cellular and molecular levels within intact living organism [2] and offered powerful methods to diagnosis illness, such as cancer, neurological diseases, cardiovascular and cerebrovascular diseases, and it contributes to improving the treatment of these disorders by optimizing the preclinical and clinical research of new medications or therapeutic regimen. Moreover, molecular imaging technologies such as positron emission tomography (PET), photon emission computed tomography (SPECT), magnetic resonance imaging (MRI), and optical imaging are applied in TCM therapy for neurological disease, which shows great potential. In this review, we focus on molecular imaging in TCM therapy for common neurological diseases. 

## 2. Traditional Chinese Medicine Therapy for Neurological Disease

TCM is one of the possible solutions utilized for the treatment of patients suffering from neurological diseases. TCM is based on the understanding that the body has an innate intelligence and healing ability [3]. The general therapeutic principle of Chinese medicine is based on its theory of “reinforcing healthy Qi and resolving and removing effects of toxicity and focusing on symptom-oriented intervention” [4]. TCM has its special advantage over western medicine in treating neurological diseases. Current studies showed that TCM can improve neural function in neurological diseases (Table 1). 

### 2.1. Chinese Herbal Medicine and Antineurological Diseases

TCM uses various vegetables, animals, and materials to emphasize treating the individual based on the principle of Zheng-syndrome differentiation of disease, aiming to restore the harmony of opposing but complementary forces [5]. Chinese herb medicine includes single herb, Chinese herbal compound, and remedies assort. The most significant characteristic of Chinese herbal compounds is that they are usually composed of multiple crude herb materials. Given that the pathogenesis and causes of most neurological diseases such as ischemic brain injury, AD, and PD could not be single factor derived, it is reasonable to use combined treatment like herbal compound with multiple biologically active components to address a variety of pathogenesis. Meanwhile, single active component extracts of the traditional Chinese herbs showed great potential in treating common neurological disease.

Bu-yang Huan-wu decoction (BHD) is one of famous TCM formulas that have been used clinically in China to treat stroke for centuries. BHD is composed of *Radix Astragali membranaceus, Radix Angelicae Sinensis, Radix Paeoniae Rubra, Rhizoma Chuanxiong, Semen Persicae, Flos Carthami, *and* Lumbricus*. Cai et al. [6] used a model of acute ischemic stroke induced by middle cerebral ischemic/reperfusion in rats and demonstrated that BHD successfully improved the neurological deficits, ameliorated the cerebral infarction, stimulate neural proliferation, and enhanced the expression of vascular endothelial growth factor (VEGF) and its receptors, which were useful for the recovery of neurological functions after ischemic stroke. Similar conclusions were obtained in the study by Wang et al.: they proved the neural protective effect of BHD by an integrative neural functional and genomic approach in ischemic stroke mice [7].

*Cornel iridoid glycoside* (CIG) is a main component extracted from the Chinese herb* Cornus officinalis*. Zhao et al. [8], using model rats with fimbria-fornix transaction (FFT), demonstrated the neuroprotective effect of CIG. In the Morris water maze and step-through test, the memory of rats in CIG (20, 60, and 180 mg/kg, resp.) treatment was significantly improved. Significant upregulation of protein level of nerve growth factor, synaptophysin, BDNF, tyrosine-specific protein kinase A, and Bcl-2 in hippocampus, while significant downregulation of cytochrome c and Bax is done, which was affected by CIG. It is indicated that CIG can protect neurons from FFT injury by promoting neuronal survival and providing a beneficial environment for brain injury repair.

*Tripterygium wilfordii Hook *(TWHF) is a traditional Chinese herb and has been historically used in TCM. The part with major pharmacological efficacy is in the root.* Tripterygium glycoside *(TII), the active anti-inflammatory component of TWHF, has been reported to be effective in therapy of many inflammatory and autoimmune diseases, such as rheumatoid arthritis and systemic lupus erythematosus and now has been used in clinical trials [9, 10]. *Triptolide*, the principal biologically active diterpenoid further purified from TII, shows good prospect in treating PD. Zhou et al. [11] confirmed the beneficial activities of* triptolide* on dopaminergic neuronal protect with an inflammatory PD model by injecting lipopolysaccharide (LPS) into the substantia nigra. After intraperitoneal injection with *triptolide* (5 *μ*g/kg) for 24 days, they found that *triptolide* significantly improved the behavior of PD rats, decreased dopaminergic neurons death, and increased dopamine level in striatum. It is indicated that *triptolide* can reduce the inflammation-mediated damage of these neurons through inhibiting the excessive release of cytokines and the overactivation of microglia induced by LPS in inflammatory PD model.

### 2.2. Acupuncture and Antineurological Diseases

Acupuncture was first reported in Yellow Emperor's Manual of Corporeal Medicine in Chinese ancient, also known as The Yellow Emperor's Inner Classic. Acupuncture has been used as a therapeutic intervention for the treatment of a variety of diseases and symptoms for more than 2500 years. However, when acupuncture was brought back by the Jesuits in the 17th century, it was a puzzle for the West. An unexpected treatment of James Reston, a famous New York Times reporter, made it popular all over the world in 1971. Since then, acupuncture was practiced in many Asian and western countries, and a diverse variety of conceptual models and styles of clinical practice and techniques have developed in this special issue [15]. According to the TCM theory, acupuncture, which was based on the principle that “functions of human whole body are controlled by the “Qi-Xue” and “Jing-Luo (meridian)” system”, has been used to balance and improve the functions of the different organs. The ancient Chinese have a profound conviction that both the universe and the human body consist of the yang and the yin. This duality of the body is expressed in the qi (yang) and the blood (yin) as two separate circulation systems. The blood is pumped by the heart and circulates through the arteries, veins, and capillaries, and the qi is generated by the lungs and flows through invisible tracts called jin-luo (meridian) in the body [16]. Nowadays, acupuncture including traditional manual acupuncture and electroacupuncture (EA), which is a significant innovation on the traditional manual acupuncture using the state-of-art technology, is a promising therapy for nervous system disorders.

To elucidate the effect of acupuncture on neurological disorders, Wang et al. [12] used EA in a mouse model of PD. They used 100 Hz EA stimulation at Zusanli (ST36) and Sanyinjiao (SP6) in 1-methyl-4-phenyl-1, 2, 3, 6-tetrahydropyridine (MPTP)-lesioned male C57BL/6 mice for 12 sessions starting from the day prior to the first MPTP injection. They found that 100 Hz EA could significantly inhibit the production of hydrogen peroxide and malonaldehyde, increase glutathione concentration and total superoxide dismutase activity, and increase the survival rate of dopaminergic neurons in substantia nigra pars compact of the MPTP-lesioned side of PD rats, which indicated that 100 Hz EA stimulation at ST36 and SP6 protects the nigrostriatal system and hinders the progressive degeneration of dopaminergic neurons by multiple mechanisms including antioxidation and anti-apoptosis. Li et al. [13] also demonstrated that acupuncture is a potential therapeutic approach for the treatment of Alzheimer's disease. They used acupuncture in male 7.5-month-old senescence-accelerated mouse prone 8 (SAMP8) mice, which was an important mouse model of aging [17]. The prescription of acupuncture points included Tanzhong (CV17), Zhongwan (CV12), Qihai (CV6), bilateral Xuehai (SP10), and bilateral Zusanli (ST36) [18, 19], and acupuncture treatment was performed once a day for 15 days in the SAMP8 acupuncture group. As a result, they found that the cognitive deficits of SAMP8 mice were improved by acupuncture treatment in the Morris water maze test, and the neuron number in hippocampal CA3 and DG of the SAMP8 acupuncture group was significantly increased by therapeutic acupuncture compared with the SAMP8 control group, which indicated that acupuncture could improve the cognitive impairment of middle-aged SAMP8 mice, attributing to the reduced neuron loss in hippocampal regions CA3 and DG, and be a effective therapy for AD. 

### 2.3. Other Nonmedication Therapies and Antineurological Diseases

Cupping, an integral part of TCM, is a physical treatment used by acupuncturists or other therapists that uses a plastic, bamboo, or glass cup to create suction on the skin over an acupuncture point or painful area [20]. It is one of the oldest medical practices and has a history of more than 2000 years in China, but varieties of it have also been used in other countries such as India, Arabia, Central Europe, and parts of Africa [21]. Usually, cupping practitioners utilize the flaming heating power to achieve minus pressure inside the cups to make them apply on the desired part of the human body. There are several major types of cupping practice such as retained cupping, bleeding cupping (or wet cupping), moving cupping, needle cupping, medicinal cupping, water cupping, and flash cupping in China [22]. Here, cupping is mainly recommended for the treatment of neurological diseases or disorders such as pain and paralysis, stroke rehabilitation and its complications, and PD.

Cupping was reported to treat stroke rehabilitation, and its complication in clinical practice has been studied by a number of researchers [23–26]. Zhang [23] evaluated the efficacy of wet cupping on responder's rate in patients with hemiplegic hand edema. Patients randomly received wet cupping and acupuncture. They demonstrated the favorable effect of wet cupping compared with acupuncture in responder's rate. After that, Park [24]tested the effects of wet cupping in patients with hemiplegic shoulder pain. A total of 58 patients were randomly divided into two groups, one receiving wet cupping plus exercise therapy and the other receiving acupuncture plus exercise therapy. After treatment, they found that the pain intensity on the visual analogue scale (VAS) and pain frequency were significantly reduced in the cupping group compared with control groups. And other two clinical trials with less sample size of patients assessed the effects of cupping for stroke rehabilitation. One trial showed that wet cupping had positive effects on aphasia after five treatments [25]. The other found that five to ten treatments of dry cupping at shenque point improved the intractable hiccup after stroke [26]. 

Cupping also has been used in treatment of PD. Cupping at back bladder meridian or channel of foot greater Yang (BL) points and governor vessel (GV) points can harmonize Qi-Blood and Yin-Yang and improve the flow of energy and blood in the body. Ding [14] revealed the effect of treatment with combined use of cupping and acupuncture for PD. 87 patients were all treated with cupping and acupuncture at 13 acupoints for 30 days, and they found that the total effective rate for PD achieved 89.66% according to the Webster scale, which demonstrated that cupping was a promising therapy for PD.

## 3. Molecular Imaging in Traditional Chinese Medicine Therapy for Neurological Diseases

With its 2,500 to 5,000-year tradition of use, TCM is one of the oldest, continuously used systems of medicine to cure a variety of diseases, particularly in neurological diseases such as ischemic brain injury, AD, and PD, because of its multi-targeted effects, less harmful side effects, high safety, and ideal effects. At the same time, in order to objectively and visually reveal the effect of TCM treatment of neurological disease, diverse molecular imaging methodologies have been applied in TCM therapy for common neurological disorders (Table 2).

### 3.1. Molecular Imaging in Traditional Chinese Medicine Therapy for Ischemic Brain Injury

Ischemic brain injury, one of the leading causes of death and adult disability all over the world, is caused by transient or permanent downregulation of cerebral blood flow initiated by arterial occlusions due to thrombotic or thromboembolic factor. Restoring cerebral perfusion timely is considered the main reasonable therapy for cerebral ischemia [27]. But reperfusion after cerebral ischemia often leads to the cascade of events including free radical-induced neuronal damage, inflammation, energy depletion excitotoxicity, apoptosis, and necrosis in cellular, biochemical, and metabolic aspects. Therefore, it is an essential task to find drugs that can effectively treat ischemic brain injury and elucidate the therapeutic mechanisms. Here, we mainly focus on applying diverse molecular imaging methodologies in different TCM therapies and monitoring the cerebral ischemia injury.

**Figure 1:**
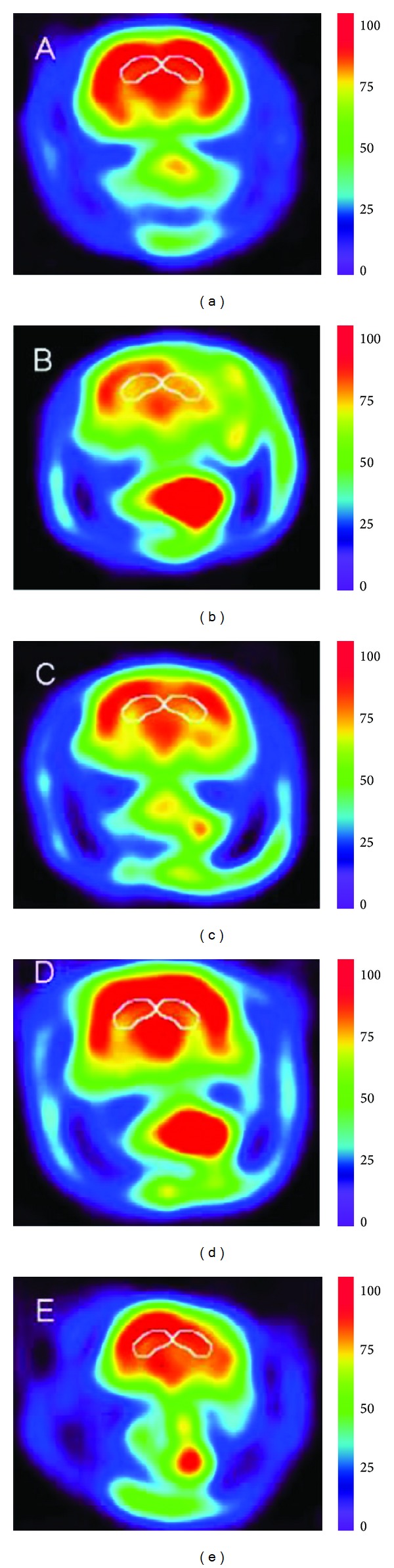
^18^F-FDG-PET images of activity of hibateral hippocampus regions glucose metabolism in rats to evaluate the effects both Astragaloside IV (ASG IV) and tetramethylpyrazine (TMPZ) on the cerebral ischemia-reperfusion injury. (a) Sham group, (b) model group, (c) ASG IV group, (d) ASG IV-TMPZ group, and (e) nimodipine group. The glucose metabolism of the ASG IV-TMPZ group was significantly recovered in the right area of cerebrum compared with other groups [31].

Micro-PET has the advantage to monitor the glucose metabolism noninvasively and assess the early effects for cerebrovascular disease therapy [28, 29], and glucose metabolism in the brain is closely related to neuronal activity [30]. Yang et al. [31] used ^18^F labeled 2-deoxy-2-fluoro-D-glucose (^18^F-FDG), as an imaging agent that reflects the state of glucose metabolism, to evaluate the effects both Astragaloside IV (ASG IV) and tetramethylpyrazine (TMPZ) on the cerebral ischemia-reperfusion injury by micro-PET. In order to uncover the therapeutic effect quantitatively, the ratio of the regions of interest (ROIs) in the temporal lobe, apical lobe, and frontal lobe to cerebellum was calculated for each group. As a result, glucose metabolism in injury brain while being treated with ASG IV and ASG IV-TMPZ was increased significantly, compared with the model group. Furthermore, glucose metabolism of the ASG IV-TMPZ group was significantly recovered in the right area of cerebrum compared with the single ASG IV group, demonstrating a visible therapeutic effect of ASG IV-TMPZ (Figure 1). Another similar study was conducted by Wan et al. [32]. They used micro-PET with ^18^F-FDG to monitor the therapeutic response of chuanxiongzine and puerarin in a rat model of transient middle cerebral artery occlusion (MCAO)-induced focal cerebral ischemia. Obvious metabolic asymmetry in the right and left hemispheres of rat after the operation of MCAO was observed, and the right hypometabolic region was enlarged distinctly in the chuanxiongzine and chuanxiongzine-puerarin groups, between which the hypometabolic region in the chuanxiongzine-puerarin group was bigger. This study represented the credible evidence that the effect of chuanxiongzine-puerarin was better than puerarin in the recovery of glucose metabolism and the infarction volume of cerebral IR damage.

As a high sensitive magnetic resonance imaging technology, diffusion weighted imaging (DWI) of MRI could measure the random translational movements of water molecules in the tissue and describe this movement results in no trauma [33], which was widely used as an accurate monitor of the lesions in the early period of cerebral ischemia. Zhang et al. [34] performed an experiment to test whether the combined administration of baicalin and jasminoidin could improve the therapeutic effect on cerebral ischemia-reperfusion injury with DWI of MRI. The result showed that apparent diffusion coefficient (ADC) value and average diffusion coefficient (DCavg) value in the peripheral zone significantly increased in the baicalin and jasminoidin combination treated group compared with that in the model group, which indicated that the therapeutic effect in cerebral ischemia injury was strongly enhanced by the combined treatment of baicalin and jasminoidin. 

Diffusion tensor imaging (DTI), a noninvasive MRI technique, measures the random motion of water molecules and provides information about cellular integrity and pathology [49]. Since 1990, it has been used to detect acute cerebral ischemia within minutes of stroke onset [50]. Wu et al. [35] investigated long-term changes of DTI after acupuncture treatment in rats with transient middle cerebral artery occlusion (tMCAO). They used the combination of Baihui (DU20), Dazhui (DU14), Shousanli (LI10), and Zusanli (ST36) as target acupoints in the treating group. As a result, particularly the fractional anisotropy value (FA) value of DTI reduced at first and increased later both in the centre and at the edge of the ischemic lesions in acupuncture group. Better recovery of FA might be due to improved neuronal regeneration induced by acupuncture treatment. Furthermore, DTI has been a helpful tool for forecasting and monitoring recovery in patients with ischemic stroke. Shen et al. [36] investigated the effects of acupuncture therapy for postponing wallerian degeneration (WD) of cerebral infarction as shown by DTI. They observed a significant difference in ADC and FA values between the acupuncture group and the control group after 8 weeks, which provided convincing evidence demonstrating the efficacy of acupuncture treatment for structural reorganization in and beyond ischemic lesions, which may contribute to functional recovery after stroke. Meanwhile Yu et al. [51] reported the same results in 2009. So we firmly believed that the dynamic evolution of WD was observed in vivo by using DTI, which may promote understanding of the effects of acupuncture treatment in stroke and might contribute to the identification of optimal strategies for stroke treatment and rehabilitation at an early stage.

Functional magnetic resonance imaging (fMRI) has also been used to evaluate the effects of acupuncture on stroke. Li et al. [37] used fMRI to assess differences in brain responses between stroke patients and controls to tactile and EA Tactile stimulations, and acupoint stimulation activated similar cortical sites, which was true both in stroke patients and normal subjects, and activation was greater in patients than controls with both tactile and electrical acupuncture stimulations. Furthermore, the intensity levels for the patients were much higher than controls due to their sensory deficits. The difference of acupoint stimulation in activation strength between patients and controls was most pronounced in the premotor cortex in a bilaterally symmetric fashion, which indicated that EA had a therapeutic effect to enhance recovery from stroke selectively activates areas thought to be involved in mediating recovery from stroke via functional plasticity. fMRI successfully illustrates the functional substrate of the purported therapeutically beneficial effect of EA in stroke rehabilitation.

As we all know, disruption of blood–brain barrier (BBB) and subsequent edema are the two major contributors to the pathogenesis of ischemic stroke. Huang et al. [38] used T2-weighted MRI, which has been considered the most promising and noninvasive approach for examining cerebral edema formation timely, confirming the brain edema reduction in Cerebralcare Granule (CG)-treated rats. CG was continuously administrated starting after 3 hours of brain reperfusion, and I/R-induced brain edema was alleviated significantly on the 6th day by T2-weighted MRI, indicating the efficiency of CG as a therapeutic strategy and a promising alternative approach for the patients at risk to develop severe brain edema. Another similar study about cerebral oedema was conducted by Zhang et al. [39]. They used DWI of MRI to determine whether EA could alleviate brain oedema after cerebral ischemia in rats. DWI showed that the relative ADC increased significantly in the cortical and subcortical areas of the EA group compared with the non-EA group, indicating that EA could alleviate cerebral oedema contributing to the treatment of ischemic stroke. 

No single imaging modality can provide all the information required to comprehensively monitor the effects of traditional Chinese medicine therapy in AD; hence, there is a requirement for combining complementary imaging methods. Indeed, the combination of PET and MRI used in the research of acupuncture [40], which has been indispensable in Chinese medicine, allows the acquisition of metabolic, anatomical, and physiological information, all from the same subject. Liu et al. [40] studied the effect of acupuncture at Baihui (GV 20) and Shuigou (GV 26) acupoints in ischemia stroke treatment of the Sprague Dawley rat animal mode by using micro-PET and DWI-MRI. They chose the FDG as an imaging agent to measure the glucose level in the brain, which is an important index of brain function [52, 53]. In order to verify the location of injured area in the brain induced by MCAO, they carried out MRI images and DWI-MRI images for 17 model rats about 12 hours after MCAO. By comparing real acupuncture with sham acupoint treatment and blank control under a simplified animal experiment setting, it was able to be verified that acupuncture indeed increased the glucose level and reduced the injury-volume in the acute stage of ischemia stroke. 

### 3.2. Molecular Imaging in Traditional Chinese Medicine Therapy for AD

AD, the most common type of senile dementia, is a neurodegenerative disorder characterized clinically by progressive memory loss and neuropathologically by extracellular amyloid plaques [54]. Nowadays, AD has become the third major cause of death to the elderly, inferior only to cardiovascular disease and cancer [55]. Since a German surgeon named Alois Alzheimer reported the first case of dementia that now bears his name in 1907, great efforts have been made in an attempt to discover effective therapy methods of AD. However, none of the current therapies such as the cholinesterase inhibitors and antagonist of N-methyl-D-aspartate receptors [56] has profound effects on halting the progression of AD, because of the complex pathological process induced by multiple factors such as oxidative stress, inflammatory responses, mitochondrial dysfunction, disturbance of energy metabolism and apoptosis [57]. TCM has been widely investigated for the treatment of AD and is regarded as promising drug candidates for AD therapy. What is more, diverse molecular imaging is applied in TCM treatment of AD, which can provide strong evidence to assess therapeutic effects and clarify therapeutic mechanisms of TCM therapy methods on AD.

PET was used to evaluate the effect of Chinese herb medicine in treatment of AD patients or AD animal models. As we all know, glucose metabolism is the primary source of energy for neurons in the central nervous system, which is considered as a useful index reflecting neural activity [41]. Therefore,^ 18^F-FDG can be used potentially as an imaging biomarker with a good sensitivity in the early diagnosis of AD [42, 58]. Yuan et al. [43] used positron emission tomography/computed tomography (PET/CT) to investigate the effect of *evodiamine* (a quinolone alkaloid from the fruit of *Evodia rutaecarpa*) on the progression of AD in SAMP8 and APP^swe^/PS1^ΔE9^  transgenic mouse models. As the AD patient exhibits large decreases in glucose uptake and energy metabolism in the frontal cortex and temporal lobes [44], they used ^18^F-FDG tracer to demonstrate the glucose uptake in brain tissue of transgenic mouse to evaluate the therapeutic effects. After 4-week administration, treatment with *evodiamine* ameliorated the glucose uptake decrease caused by APP^swe^/PS1^ΔE9^  expression by 16%. That is to say, *evodiamine* significantly improved the glucose uptake and cognitive abilities in the APP^swe^/PS1^ΔE9^  transgenic mice, to some extent which suggested that *evodiamine* could have potential usage in treatment of AD. Li et al. [45] had used the micro-PET with ^18^F-FDG as the tracer to study the effect of *Fuzhisan* (FZS), a Chinese herbal complex prescription, on the naturally aged rats. The result showed that the decreased ^18^F-FDG uptake in the temporal and parietal cortices of the aged rats was improved significantly by FZS treatment for 30 days, which implied that the amelioration of the glucose metabolism in brains of the aged rats treated with FZS may be another important mechanism of the FZS therapy for AD. PET was also used in clinical research. Bi et al. [41] took advantage of ^18^F-FDG-PET to investigate the effects of FZS (10 mg/day) on cerebral glucose metabolism in patients with mild-to-moderate AD. In order to objectively elucidate the theraputic efficacy of FZS in treatment of AD patients, the regional cerebral metabolic rate of glucose consumption (rCMRglc) at baseline and week 12 was taken into account by using PET. The result showed that FZS significantly increased rCMRglc in the bilateral temporal and parietal cortices, hippocampus, and posterior cingulate gyrus, which indicated that elevation of rCMRglc is an important index of the mechanism mediating the effects of FZS in treatment of AD. Therefore, we have reason to believe that ^18^F-FDG-PET may become a useful tool in evaluating pharmacotherapeutic treatment responses in AD with traditional Chinese medicine.

**Figure 2:**
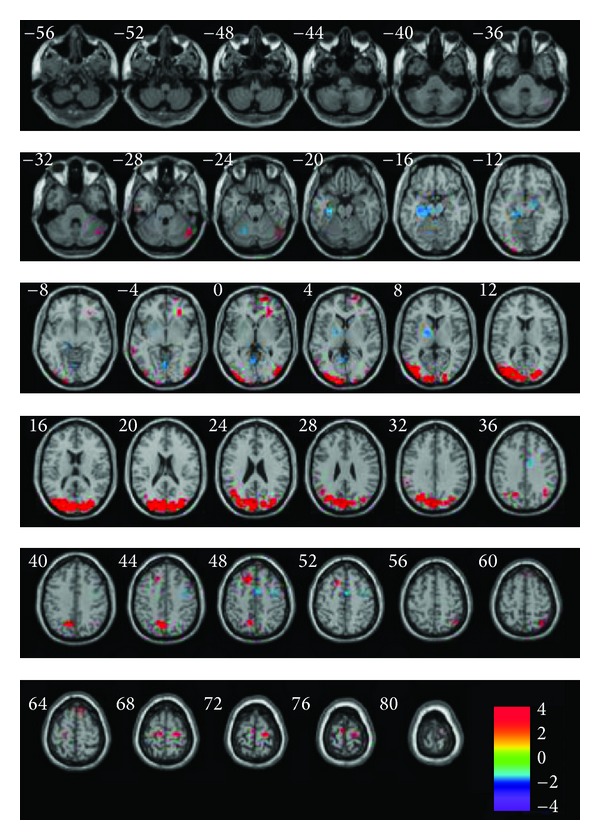
fMRI images of regions showing increased or decreased activities in entire brain of Alzheimer's disease patients after acupuncture compared with the resting state. Left in picture is left in the brain. The color scale represents *t* values [60].

fMRI was used clinically to investigate the effect and clarify the mechanisms of acupuncture in treating AD. Zhou and Jia [59] explored various regions of the brains of AD patients before and after acupuncture treatment of Shenmen (HT7), Zusanii (ST36), Fenglong (ST40), and Taixi (KI3) acupoints by using fMRI. The result demonstrated that there were left activated regions (temporal lobe, parietal lobule, and some regions of cerebellum) and right main hemisphere activations (temporal lobes, such as hippocampal gyrus, insula, and some area of parietal lobe), both of which were induced by these acupoints. To our surprise, the activated region, closely correlated with the cognitive function, consisted of the impaired areas in brain for AD patients. In order to better understanding of the pathophysiology of AD, Wang et al. [60] attempted to investigate the effect of acupuncture at the acupoints of Tai-chong (Liv3) and Hegu (LI4) in left and right sides on the brain functional activity throughout the entire brain in AD patients compared with normal controls. The result showed the increased activities in the regions of right cerebellum posterior lobe, bilateral frontal lobe, right inferior parietal lobule, and right middle occipital lobe, and the decreased activities in the regions of right superior temporal gyrus, right middle temporal gyrus, bilateral middle frontal gyrus, and left brain stem from that of resting state in the process of acupuncture. Posteffect of the acupuncture was further examined, and the activated regions included the frontal lobe, the occipital lobe, the parietal lobe, and the temporal lobe (Figure 2). They speculated that the temporal lobe, as is subjected to be impaired in AD patients, was activated to compensate for the cognitive impairment. These present studies using fMRI provided the strong evidence that acupuncture had a potential effect on AD.

### 3.3. Molecular Imaging in Traditional Chinese Medicine Therapy for PD

PD is one of the most common neurodegenerative disorders, second in prevalence only to AD, and affects about 1% to 2% of the elderly over the age of 60 [61]. The pathological characteristic of PD is a progressive loss of dopaminergic neurons in substantia nigra of midbrain, followed by the significantly decreased content of dopamine as the neurotransmitter in striatum and resulting in the clinical symptoms [46]. The initial description of PD was made by James Parkinson in 1817, which was accepted in western medical system. However, TCM has played an important role in the treatment of patients with PD for thousands of years in China. The precise records for the symptoms of PD and its primary therapy prescriptions could date back to the Eastern Han Dynasty (206 BC-220 AD) [62]. Moreover, the first time describing a typical case of PD was made by Zhang Zihe (1156-1228 AD) in his book *Confucian's Duties to Their Parents*, which was recorded 600 years earlier than those reported by James Parkinson [47]. Nowadays, molecular imaging such as PET and SPECT, representing new modern technologies, was applied in TCM therapy for PD to evaluate the effect of TCM on PD treatment.

PET was used clinically to study the effect of acupuncture in treatment of PD patients. Huang et al. [48] used PET and ^18^F-FDG tracer to study cerebral effects of complementary acupuncture in PD patients. The PET images demonstrated that complementary acupuncture increased regional cortical glucose metabolism bilaterally in parietal and occipital lobes and, in the temporal lobe, the cerebellum and the thalamus of the least-affected side, compared with Madopa-only group. Hence, complementary acupuncture may improve cerebral glucose metabolism in Parkinson's disease.

**Figure 3:**
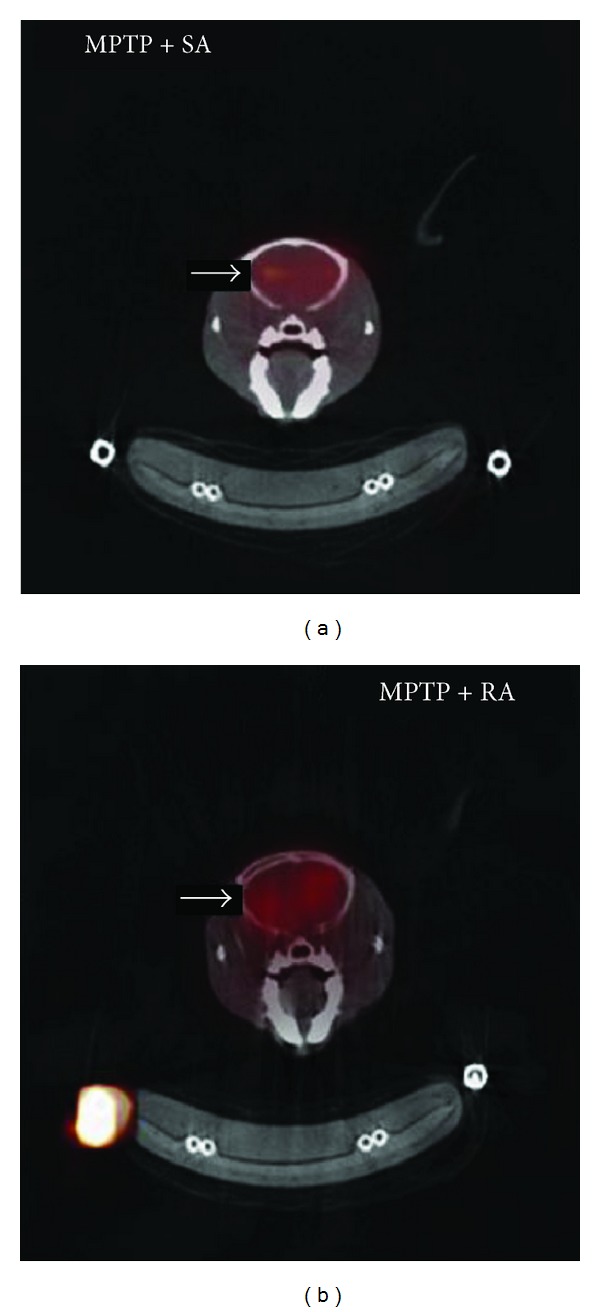
[^123^I]IBZM-SPECT images of the acupuncture-treated MPTP-induced Parkinson's disease mice. The left is sham acupuncture (SA) group, and the right is retained acupuncture (RA) group. The intensity of radionuclide or radiopharmaceutical uptake in RA group is higher than that in SA group [65].

SPECT is a technique that uses a tracer to acquire images that reflect fundamental biophysiologic functions of perfusion and metabolism in different body organs, by analysing the temporal changes of radionuclide concentration in tomographic sections through angular sampling of projections [63]. SPECT was used to reveal the effect of acupuncture in treatment of PD patients or PD animal models. Huang et al. [64] investigated cerebral effects of complementary acupuncture in PD patients by using SPECT with ^99m^Tc-ECD and ^99m^Tc-TRODAT-4, both before and after five weeks of treatment. The result showed that combination acupuncture and levodopa increased regional cerebral blood flow (rCBF) in the frontal lobe, the basal ganglion, the occipital lobe, and the cerebellum in the most affected hemisphere as compared with baseline, whereas there were no changes in basal ganglia dopamine transporter (DAT) levels. Thus, complementary acupuncture treatment in PD may affect rCBF but not basal ganglion DA. Another study about acupuncture in PD animal modes also used SPECT. Yang et al. [65] investigate the role of retained acupuncture (RA) in neurotoxin-induced PD mice with [^123^I] IBZM-SPECT imaging. The SPECT imaging showed that the intensity of radionuclide or radiopharmaceutical uptake in RA group is higher than that in sham acupuncture (SA) group (Figure 3). By using quantitative analysis, the peak time of [^123^I] IBZM uptake was longer than RA group, which suggesting that the delayed kinetic change by MPTP damage could be reversed by RA treatment. Therefore, RA may be useful as a complementary strategy when treating PD.

## 4. Summary and Perspectives

TCM including Chinese herbal medicine, acupuncture, and other nonmedication therapies may offer a unique strategy in combating the devastating neurological diseases. A variety of molecular imaging methodologies, such as PET, SPECT, and MRI, have been applied in TCM therapy for neurological disorders, which shift away from classical morphological measures towards the assessment of functional, cellular, metabolic, and molecular information in vivo [66]. In addition, diverse molecular imaging objectively and visually reveals the effect of TCM treatment and clarifies the therapeutic mechanisms of different types of neurological disease.

 However, more effective treatment is critically required not only for ameliorating the clinical symptoms but also slowing down or reversing of the progress of the disease. Thus, it is still far from thoroughly verifying the effectiveness of TCM in neurological diseases treatment, and it shows that we can probe the consequences of TCM quantitatively with a target specific imaging technique. As TCM in general has the reputation of being mysterious, our review offers an example of how TCM therapy in neurological disorders can be explained with modern scientific language.

In the future, we also could take advantage of molecular imaging, especially PET imaging, to investigate the pharmacokinetics study of TCM. We choose some biological active components of Chinese herb medicine, which are similar to the tracers of PET in molecular structure. Later, the biological active component, as a tracer, is labelled with a positron emitting isotope, such as ^11^C, ^18^F, and ^15^O [67]. For example, *salvianic acid A* is one of the most effective water-soluble components in *Danhong injection*, which has been widely used in treating cardiovascular and cerebrovascular diseases [68]. The molecular structure of *salvianic acid A* is similar to FDG, and it could be labeled with ^18^F. Then, we can use PET with [^18^F]-*salvianic acid A* as a tracer to study its dynamic biodistribution of in the brain and the correlation of its concentration and the changes in brain function.

In conclusion, we believe that a combination of TCM and modern molecular imaging techniques will initiate new approaches for neurological diseases treatment.

## Figures and Tables

**Table 1:** Traditional Chinese medicine therapy in neurological disease models of animal or human.

TCM type	Drug or acupoints	Disease models	Therapy time (days)	Functional outcome	References
CHM	BHD	MCAO (rats)	14	Enhanced neurological recovery	[6]
		CI/R (mice)	14	Improved functional recovery	[7]
	CIG	AD (rats)	28	Promoted neuronal survival and brain repair Attenuated neurological symptoms	[8]
	Triptolide	PD (rats)	24	And inflammatory reactivity	[11]

ACU	ST36, SP6	PD (mice)	13	Protected the nigrostriatal system	[12]
	CV6, 12, 17, SP10, ST36	AD (mice)	15	Improved cognitive deficits, reduced neurons loss	[13]

CUP	BL, GV	PD (human)	30	Attenuated neurological symptoms	[14]

CHM: Chinese herb medicine; ACU: acupuncture; CUP: cupping; BHD: Bu-yang Huan-wu decoction; CIG: cornel iridoid glycoside; ST36: Zusanli; SP6: Sanyinjiao; CV6: Qihai; CV12: Zhongwan; CV17: Tanzhong; SP10: bilateral Xuehai; BL: Bladder meridian or channel of foot greater Yang; GV: governor vessel; MCAO: middle cerebral artery occlusion; CI/R: middle cerebral ischemic/reperfusion; AD: Alzheimer's disease; PD: Parkinson's disease.

**Table 2:** Molecular imaging in traditional Chinese medicine therapy for neurological disease.

Disease models	CHM type	Drug or acupoints	Tracers	Modality	Functional outcome	References
MCAO (rats)	CHM	ASG IV-TMPZ	^ 18^F-FDG	PET	Enhanced functional recovery and glucose metabolism	[31]
MCAO (rats)	CHM	CXQ-PUE	^ 18^F-FDG	PET	Recovery glucose metabolism, reduced infarction volume	[32]
MCAO (rats)	CHM	BAI-JAS	NULL	DWI-MRI	Enhanced cerebral ischemia injury repair	[34]
MCAO (rats)	ACU	DU14, 20 LI10, ST36	NULL	DTI-MRI	Improved neurons regeneration	[35]
WD (human)	ACU	Du20, 23, EXH- N3, PC6, Sp6	NULL	DTI-MRI	Improved functional recovery	[36]
Stroke (human)	ACU	LI 14, LI11	NULL	fMRI	Enhanced functional recovery	[37]
Stroke (human)	CHM	CG	NULL	T2W-MRI	Ameliorated brain edema	[38]
MCAO (rats)	ACU	GV20	NULL	DWI-MRI	Alleviated brain oedema	[39]
MCAO (rats)	ACU	GV20, GV26	^ 18^F-FDG	DWI-MRI and PET	Improved metabolic recovery, reduced the injuryvolume	[40]
AD (mice)	CHM	Evodiamine	^ 18^F-FDG	PET/CT	Improved glucose uptake and cognitive abilities	[41]
AD (rats)	CHM	Fuzhisan	^ 18^F-FDG	PET	Ameliorated glucose metabolism of brain	[42]
AD (human)	CHM	Fuzhisan	^ 18^F-FDG	PET	Increased rCMRglc	[43]
AD (human)	ACU	ST36, 40, HT7, KI3	NULL	fMRI	Improved cognitive function	[44]
AD (human)	ACU	Liv3, LI4	NULL	fMRI	Enhanced cognitive recovery	[45]
PD (human)	ACU	MS4, 6, 9, 14	^ 18^F-FDG	PET	Improve cerebral glucose metabolism	[46]
PD (human)	ACU	MS4, 6, 8, 9, 14	^ 99m^Tc-E, ^9m^Tc-T4	SPECT	Increased rCBF	[47]
PD (mice)	ACU	PC 7	[^123^I]-IBZM	SPECT	Attenuated neuronal damage	[48]

MCAO: middle cerebral artery occlusion; WD: wallerian degeneration; AD: Alzheimer's disease; PD: Parkinson's disease; CHM: Chinese herb medicine; ACU: acupuncture; ASG IV: Astragaloside IV; TMPZ: tetramethylpyrazine; CXQ-PUE: chuanxiongzine and puerarin; BAI-JAS: baicalin and jasminoidin; DU20: Baihui; DU14: Dazhui; LI10: Shousanli; ST36: Zusanli; Du 23: Shangxin; EXHN3: Yintang; PC6: Neiguan; Sp6: Sanyingjiao; LI11: Quch; LI14: Binao; CG: cerebralcare granule; GV20: Baihu; GV26: Shuigou; HT7: Shenmen; ST36: Zusanii; ST40; Fenglong; KI3: Taixi; Liv3: Taichong; LI4: Hegu; MS6: the anterior oblique meridian of the vertex to temple; MS4: the lateral III meridian on forehead; MS8: the lateral I meridian of the vertex; MS9: the lateral II meridian of the vertex; MS14: lower-lateral meridian of the occiput; MS8: the lateral I meridian of the vertex; PC: Daling; [^18^F] FDG: 2-deoxy-2-(^18^F)fluoro-D-glucose; ^99m^Tc-E ^99m^Tc-ECD: technetium-99m ethyl cysteinate dimer; ^99m^Tc-T4: ^99m^Tc-TRODAT-4; [^123^I] IBZM: ^123^I-iodobenzamide; rCMRglc: regional cerebral metabolic rate of glucose consumption; rCBF: regional cerebral blood flow.
